# Comparative clinico-pathological observations in young Zebu (*Bos indicus*) cattle experimentally infected with *Trypanosoma vivax* isolates from tsetse infested and non-tsetse areas of Northwest Ethiopia

**DOI:** 10.1186/s12917-015-0625-0

**Published:** 2015-12-23

**Authors:** Shimelis Dagnachew, Getachew Terefe, Getachew Abebe, Asegedech Sirak, Enrico Bollo, Dave Barry, Bruno Goddeeris

**Affiliations:** Addis Ababa University, College of Veterinary Medicine and Agriculture, Debre Zeit, Ethiopia; Food and Agriculture Organization of the United Nations, Addis Ababa, Ethiopia; National Animal Health and Disease Investigation Center, Sebeta, Ethiopia; University of Turin, DSV, Turin, Italy; University of Glasgow, CMVLS, Glasgow, UK; Catholic University of Leuven, Faculty of Bioscience Engineering, Leuven, Belgium

**Keywords:** Cattle, Clinical findings, Gross lesions, Histopathological lesions, *Trypanosoma vivax*, Northwest Ethiopia

## Abstract

**Background:**

The Northwest region of Ethiopia is affected by both tsetse and non-tsetse transmitted trypanosomosis with a huge impact on livestock productivity. The objective of this experimental study was to determine clinical and pathological findings in young Zebu cattle experimentally infected with *Trypanosoma vivax* isolates from tsetse infested and non-tsetse infested areas of Northwest Ethiopia. A total of 18 cattle (*Bos indicus*) aged between 6 and 12 months, purchased from a trypanosome-free and confirmed to be trypanosome negative divided into three groups of six animals were used. Animals in the first two groups (Group TT: tsetse infested isolate infected and Group NT: non-tsetse infested isolate infected) received 2 mL of infected blood from donor animals at 10^6^ trypanosomes/mL, and the remaining group was non-infected control (NIC). Each group was observed for a period of eight consecutive weeks, daily for clinical signs and once per week for parasitaemia. Postmortem examinations were done on euthanized animals, and tissue samples were taken for histopathological analysis.

**Results:**

The prepatent period of the disease was earlier in the NT group 6 days post infection (dpi) than TT group 12 dpi. The infection was characterized by reduced feed intake, intermittent pyrexia and parasitaemia, enlarged lymph nodes, lacrimation, reduced feed intake and emaciation. Less frequently diarrhea, oedema and nervous signs were observed in both groups of infected animals. At necropsy, infected animals showed enlarged spleen, enlarged lymph nodes, pneumonic and emphysematous lung, enlarged liver, and haemorrhages on the brain and intestine. Histopathological analysis revealed lymphoid hyperplasia of the spleen, necrosis of the liver, encephalitis and hyperplasia of lymph nodes.

**Conclusion:**

*Trpanosoma vivax* isolates from both tsetse infested and non-tsetse areas showed a variety of virulence factors leading to the development of acute clinical signs, gross and histopathological lesions. However, the parasitaemia and clinical signs appeared earlier in the NT compared to TT infected groups.

## Background

Trypanosomosis is a parasitic disease caused by different species of flagellated protozoa belonging to the genus *Trypanosoma* which inhabit the blood, various body tissues and fluids of vertebrate host. However, the extent of tissue invasion varies among the different species of the parasite [1, 2]. It is frequently fatal and is a serious constraint to agricultural production in large parts of sub-Saharan Africa. It has direct impacts on livestock productivity, livestock management and human settlement, and indirect impacts on crop agriculture and human welfare [3]. Trypanosomosis is transmitted cyclically by tsetse flies, the principal vector of trypanosomes. However as some species of trypanosomes are transmitted by other biting flies by non-cyclical (mechanical) transmission, the success of tsetse eradication programmes may be compromised. Several tabanids and other biting fly species have proven to be mechanical vectors of trypanosomes [4]. Trypanosomosis is prevalent in two main regions of Ethiopia i.e., the Northwest and the Southwest regions [5–8]. The most important trypanosomes in the country are *T. vivax*, *T. congolense* and *T. brucei. Trypanosoma vivax* is the most common species found in tsetse infested and non-tsetse infested areas of Ethiopia, while *T. congolense* is the common species in tsetse infested areas [9]. Both species affect a large number of cattle which are the most important species of domestic animals in Ethiopia. The wide spread of *T. vivax* is due to its adaptation to mechanical transmission by biting flies in areas outside tsetse fly belt [5, 8, 9].

The pre-patent period of bovine trypanosomosis is usually 1 to 3 weeks, depending on the virulence of the infecting trypanosome, the infective dose and the immune status of the host. Experimental infection with African trypanosomes typically follows three successive stages: acute, stabilization and chronic. The early acute phase of the disease is characterized by the continuous presence of trypanosomes in the blood at detectable concentrations (10^3^-10^8^/mL) [10]. Fever is highest at the first peak of parasitaemia and fluctuates thereafter with parasitaemia waves. With the onset of parasitaemia, anaemia develops. Clinical signs associated with trypanosomosis include pallor of the mucous membranes, lymph node and spleen enlargement, weakness, lethargy, loss of condition, abortion and reduced milk production [11]. The main haematological changes observed in natural cases of bovine trypanosomosis due to *T. vivax* infections are anaemia associated with decrease in packed cell volume (PCV), hemoglobin and red blood cell (RBC) counts, and severe leukopenia. The leukocyte changes are characterized by relative lymphocytosis and monocytosis and a decrease in neutrophil counts [12]. Similarly, blood cellular damage and histopathological changes in liver, kidney, spleen and other related organs were found in cattle experimentally infected with trypanosomes [13]. Information related to the impact of *T. vivax* infections on haematological values as well as clinico-pathological abnormalities is scarce in Ethiopia. For the purpose of better understanding the pathogenicity of *T. vivax* in tsetse and non-tsetse infested areas of Northwest Ethiopia, the present study was therefore carried out to determine and compare the PCV and differential white blood cell (WBC) counts, clinical and pathological findings induced by *T. vivax* isolates from tsetse and non-tsetse infested areas of Northwest Ethiopia in experimentally infected young Zebu cattle.

## Methods

### Experimental animals

Eighteen young Zebu (*Boss indicus*) cattle aged between 9 and 12 months were purchased from Debre Brehan, which is located in a trypanosome free area of northcentral highland Ethiopia. Before transportation animals were treated with long acting oxytetracycline (Alamycin LA, Norbrook, Ireland). On arrival at the College of Veterinary Medicine and Agriculture of Addis Ababa University, the animals were ear-tagged and screened for haemoparasites along with internal and external parasites. Animals were dewormed with albendazole (Albenda-QK, Chengdu Qiankun, China), and ivermectin (Ivermectin 1 %, Chengdu Qiankun, China) for the control of internal and external parasites. The animals were housed a in a fly proof experimental animal facility at the College of Veterinary Medicine and Agriculture of Addis Ababa University from November 2012 - March 2013. Prior to the beginning of the experiment the animals were subjected for one month acclimatization period for the new environment, handling and feeding conditions.

### Feeding and animal management

Throughout the experiment animals were fed *ad libitum* with grass hay and water, and supplemented with concentrates, green elephant grass and mineral licks. The handling of animals during the experiment was based on international guiding principles for biomedical research involving animals proposed by the Council for International Organizations of Medical Sciences [14]. The research was authorized by the Animal Research Ethics Review Committee of the College of Veterinary Medicine and Agriculture of the Addis Ababa University (Permit No: VM/ERC/003/04/013). At the end of the experiment, all of the infected animals were euthanized using overdose sodium phenobarbitol injection via jugular vein for gross pathological and histopathological examination and subsequent disposal. Similarly animals suffering with severe clinical manifestation were euthanized before the end of the experiment, and examined for pathological analysis.

### Experimental groups

The experimental animals were divided randomly into three groups. Group I (TT) was infected with *T. vivax* isolate from tsetse-infested area, Group II (NT) was infected with *T. vivax* isolate from non-tsetse infested area and Group III (NIC) was the non-infected controls. Each group was kept in a separate pen.

### Trypanosome challenge

*Trypanosoma vivax* isolates originated from naturally infected cattle of Northwest Ethiopia: one isolate from a tsetse infested area (Jabitehenan district of Birsheleko area-ETBS 1) and one isolate from non-tsetse infested area (Bahir Dar Zuria district- ETBD 1) about 300 km from the first area. The isolates were confirmed pure *T. vivax* by PCR [15]. Furthermore the non-tsetse *T. vivax* isolate (ETBD 1) was sequenced using Proline racemase PCR [16] and found to be very similar to *T. vivax* isolates from West African, South American and other regions of Ethiopia. The parasitaemia in the donor animals was estimated according to the “rapid-matching method” described by Herbert and Lumsden [17] for the experimental infections. Each experimental animal received 2 mL of infected blood from the donor animal at 10^6^ trypanosomes/mL by intravenous route.

### Parasitological and clinical examinations

Animals were examined daily for clinical parameters at their pen during the study period. Visible mucus membrane, palpable lymph nodes, skin elasticity and other conditions were thoroughly inspected. Rectal temperature was taken twice weekly in the morning with a digital thermometer. Blood samples were examined for the presence of trypanosomes daily until the detection of parasites, then weekly after parasitaemia was established using wet blood smears and buffy coat technique [10, 18].

### PCV determination and differential WBC count

Blood samples collected in EDTA vacutainer tubes were used for PCV determination and differential WBC count. PCV was measured by haematocrit centrifugation technique using a Hawksley microhaematocrit reader. Thin blood smear were prepared with Giemsa stain for differential leukocyte counts, based on 100 cells per slide according to their staining reactions, shape of the nucleus, and presence or absence of granules in their cytoplasm [19].

### Gross and histopathological examination

Four animals showing severe clinical signs, and all animals at the end of the experiment were humanely euthanized using an overdose 20 % phenobarbitol sodium intravenous administration. Gross pathological changes such as change in size, weight, color, consistency and texture were noted on each examined organ. Representative tissue samples were taken from lymph nodes, spleen, liver, heart, kidney, lung and brain, and fixed in 10 % neutral buffered formalin for histopathological studies. Formalin fixed tissues were dehydrated in ascending grades of alcohol, cleared by two changes of xylene, paraffin embedded, sectioned at 3–5 μm thickness, and stained with haematoxylin and eosin for microscopic examinations [20]. At high magnification, cellular changes such as macrophage and leucocyte infiltrations, cell and tissue damages were recorded.

### Data analysis

The entire data was entered to Excel spreadsheet and imported to SPSS version 20 for statistical evaluation. Descriptive statistics was used to explain the clinico-pathological findings. Differences in mean PCV, rectal temperature and differential WBC count measured between groups were analyzed by Two-Way repeated measures ANOVA. Significant level was set at *P* < 0.05.

## Results

### Parasitaemia and clinical findings

**Fig. 1:**
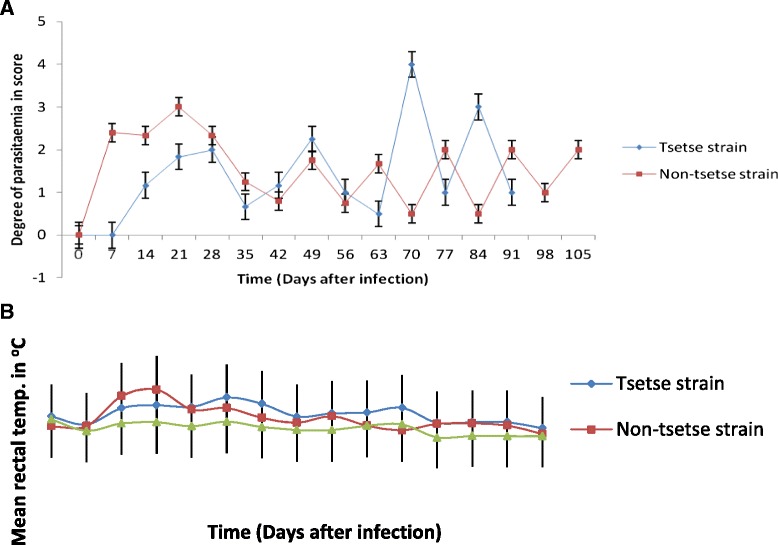
Mean waves of parasitaemia and rectal temperature in *T. vivax* experimental infections of cattle. **a** Mean waves of parasitaemia in *T. vivax* experimental infections of young Zebu cattle with (TT-tsetse strain, NT-non-tsetse strain and NIC-non-infected control groups) examined daily for the first 14 days and weekly until the end of the study; the degree of parasitaemia score was estimated based on the number of parasites per microscopic field. **b** Mean ± SD rectal temperature in *T. vivax* experimental infections of young Zebu cattle (TT-tsetse strain, NT-non-tsetse strain and NIC-non-infected control groups) measured in 2-4 days interval during the study period; the maximum peak was seen at 10 dpi for non-tsetse isolate, and at 18 dpi for tsetse isolate

Parasitaemia was detected at the sixth day post-infection (dpi) in animals infected with *T. vivax* isolated from non-tsetse infested area, and at the twelfth dpi in animals infected with tsetse infested isolate. Animals in group NT showed early peak parasitaemia on day 8 compared to group TT which showed on day 14. Animals remained trypanosome positive with fluctuating parasitaemia throughout the experimental period (Fig. 1a). The mean rectal temperatures of infected groups (39.07 ± 0.64, 39.17 ± 0.70 for TT and NT respectively) were significantly higher (*p* < 0.001) than that of the non-infected controls (38.39 ± 0.30). However, no significant difference was found among infected groups. The temperatures of infected animals started rising from the fifth dpi coinciding with the appearance of parasitaemia. The rise in temperature was followed by fluctuations (Fig. 1b). The highest mean temperature recorded during the study was 40.5 °C in NT group on the tenth dpi.

**Fig. 2:**
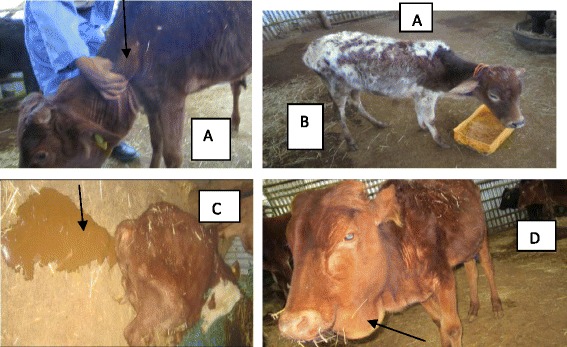
Major clinical findings in cattle experimentally infected with TT and NT *T. vivax* isolates. **a** Enlarged prescapular lymph node (arrow) at 12 dpi in TT (tsetse infested isolate) infected group; **b** emaciation at 21 dpi in NT (non-tsetse infested isolate) infected group; **c** diarrhea (arrow) at 12 dpi in NT infected group; **d** oedema at 19 dpi in TT infected group

All the infected cattle developed acute trypanosomosis, characterized by different clinical signs. Fever, staring hair coat, enlarged superficial lymph node (Fig. 2a), congested mucus membrane, lacrimation, reduced feed intake and rapid weight loss were early clinical findings, whereas pallor of mucus membrane, dehydration, and emaciation (Fig. 2b) were predominant in the later stage of the infection. Less frequent signs seen include dullness, diarrhea (Fig. 2c), oedema (Fig. 2d), corneal opacity, weakness and recumbence. Four infected animals (two from TT and another two in NT groups) showed severe clinical manifestations (PCV below 15 %, nervous signs and recumbency) were euthanized using an overdose phenobarbitol sodium intravenous administration at the 28^th^ and 30^th^ day post-infection (dpi).

### PCV values and differential WBC count

**Fig. 3:**
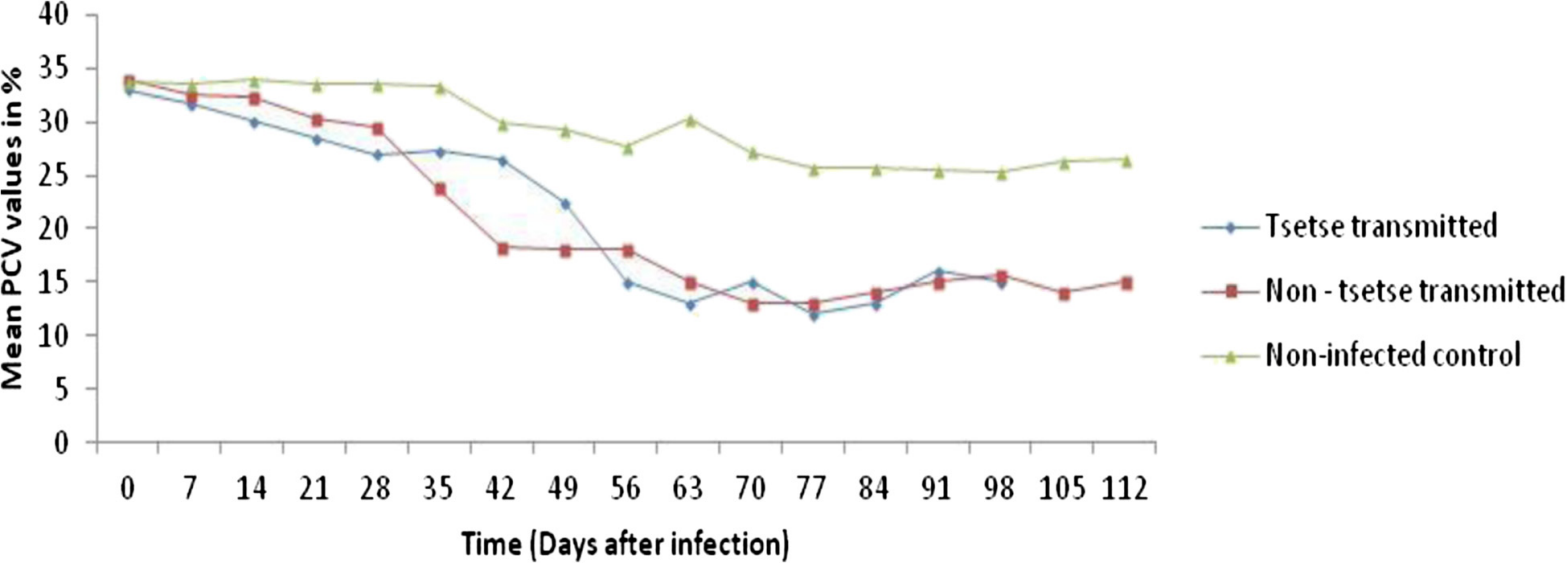
Mean PCV values in cattle experimentally infected with *Trypanosoma vivax* isolates. Mean ± SD PCV values in young Zebu cattle experimentally infected with *T. vivax* isolates from tsetse infested (TT) and non-tsetse infested (NT) areas of Northwest Ethiopia and non-infected control (NIC) groups during the study period

A significant decrease (*p* < 0.001) in the mean PCV was detected in all infected groups (22.72 ± 3.55, 20.41 ± 3.67 for TT and NT, respectively) compared to the non-infected control group (27.19 ± 2.05). Mean PCV values of infected groups was decreased gradually and reached significant levels on day 14 p.i. for NT and on day 21 p.i. for TT groups (Fig. 3).

The mean differential leukocyte counts in the infected groups was decreased compared to that of the non-infected control levels between 14 and 21 dpi for TT and NT and remained below that of the non-infected control group until days 49 and 56, respectively. The basophil counts in all the infected groups fluctuated within the control levels throughout the experiment. Lymphocyte, neutrophil and eosinophil levels were depressed in infected groups (*p* < 0.001). Monocyte count shows slight but not significant elevation (Table 1).

**Table 1:** Mean differential WBC counts during the study period (mean of recordings of 8 consecutive weeks) in young Zebu cattle experimentally infected with *T. vivax* isolates from tsetse infested (TT) and non-tsetse infested (NT) areas and non-infected control (NIC) groups

Differential WBC count	Group	Mean ± SD	95 % CI^a^ for mean	*P-* value
Lymphocyte count (x10^3^/μl)	TT	4.01 ± 1.44	3.26-4.33	0.000
	NT	4.00 ± 1.07	3.72-4.50	
	NIC	5.66 ± 0.94	5.22-5.23	
Monocyte count (x10^3^/μl)	TT	0.59 ± 0.35	0.46-0.71	0.034
	NT	0.57 ± 0.28	0.47-0.68	
	NIC	0.49 ± 0.20	0.42-0.56	
Neutrophil count (x10^3^/μl)	TT	1.84 ± 1.00	1.48-2.20	0.000
	NT	2.14 ± 1.44	1.62-2.67	
	NIC	2.60 ± 0.77	2.34-2.86	
Eosinophil count (x10^3^/μl)	TT	0.20 ± 0.22	0.11-0.28	0.000
	NT	0.18 ± 0.11	0.15-0.23	
	NIC	0.40 ± 0.23	0.32-0.48	
Basophil count (x10^3^/μl)	TT	0.04 ± 0.05	0.02-0.06	0.573
	NT	0.02 ± 0.04	0.00-0.04	
	NIC	0.02 ± 0.04	0.01-0.03	

95 % CI^a^ -95 % confidence interval

### Gross pathological analysis

**Fig. 4:**
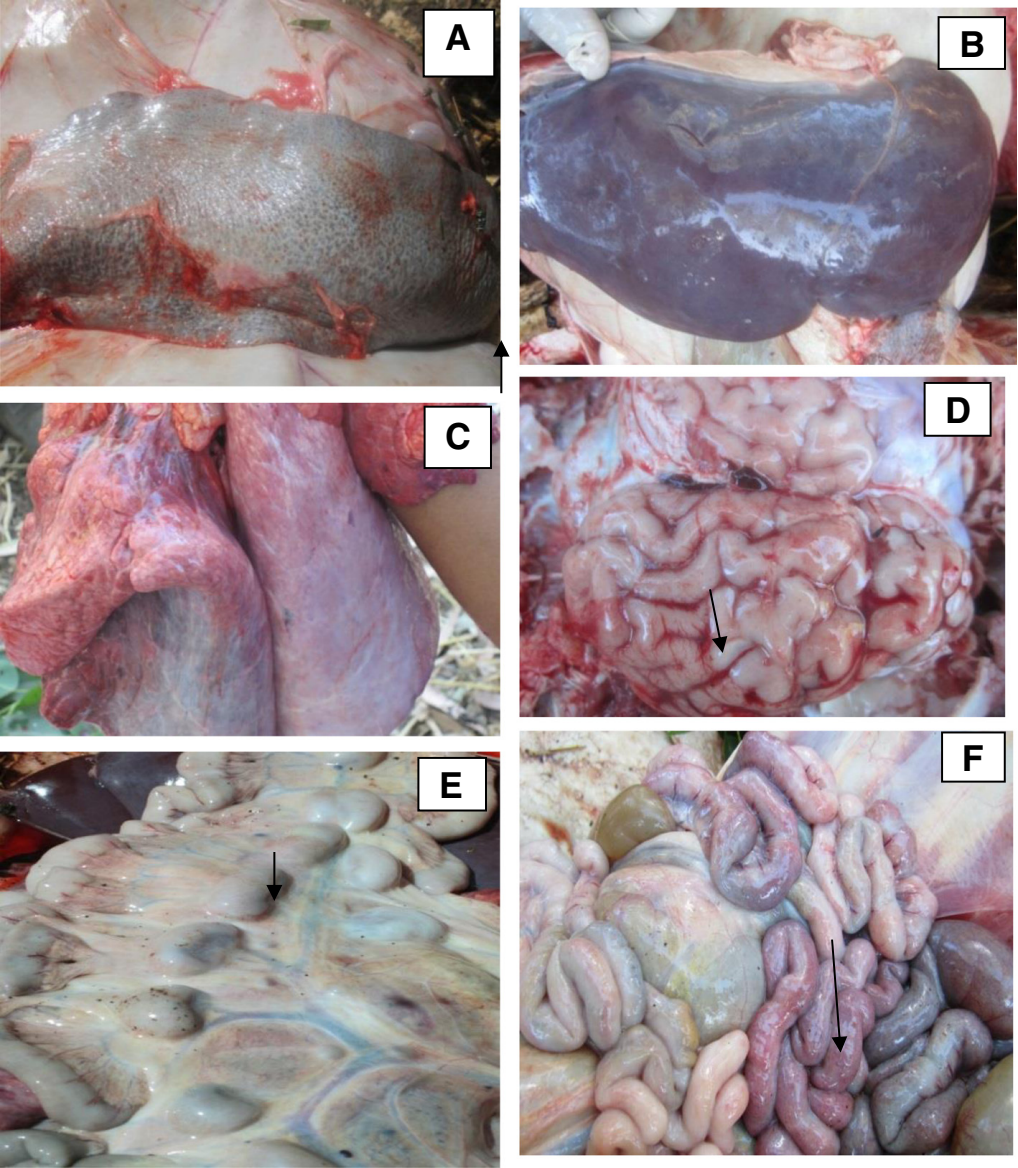
Major gross pathological findings in young Zebu cattle experimentally infected with *T. vivax* isolates. **a** Haemorrhagic and enlarged spleen with rounded edge (arrow); **b** enlarged and edematous liver with rounded edge and focal necrotic lesions; **c** lung showing emphysema and bluish discoloration on lobular areas; **d** edematous and haemorrhagic brain (arrow); **e** enlarged and edematous mesentric lymph nodes (arrow); **f** haemorrhagic and oedematous intestine

The major gross pathological findings were: swollen and edematous lymph nodes, highly enlarged spleen, enlarged and congested liver, adhesion of kidney with liver, petechiation of the peritoneum, haemorrhages and congestion on the pericardium and the brain, and emaciated carcass. The most common gross lesions include splenomegaly with hemorrhagic spleen (Fig. 4a), enlarged liver covered with fibrinous exudates and white spot lesions (Fig. 4b), pneumonia with bluish discoloration of the lungs (Fig. 4c), oedema and focal hemorrhages and meningitis in the brain (Fig. 4d), and lymphadenopathy with enlarged, haemorrhagic and edematous lymph nodes (Fig. 4e). Other significant and frequent lesions in infected animals were widespread ecchymotic hemorrhages of the small intestine (Fig. 4f), and petechial haemorrhages in the kidney and heart. The post mortem examination of the animals in the control group did not reveal any significant gross lesion.

### Histopathological analysis

#### Lymph node

**Fig. 5:**
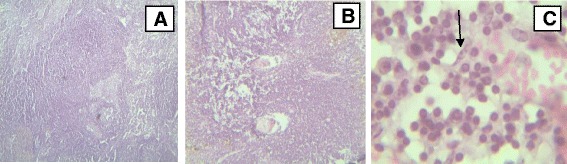
Major histopathological findings in lymph nodes of cattle experimentally infected with *T. vivax* isolates. **a** Enlargement of lymphatic follicles and plasma cell infiltration (low magnification); **b** severe lymphoid hyperplasia which forms lymphoid cords in the medullary region of the lymph node (low magnification); **c** severe infiltration of plasma cells and presence of parasites (arrow) (high magnification), (haematoxylin and eosin staining)

The most frequent and common lesions of lymph nodes in infected animals were marked lymphoid hyperplasia with prominent germinal centers. Marked increases of large lymphocytes and plasma cells, and lymphoid depletion with formation of cavity in the lymphoid follicles of cortex in some animals were detected (Fig. 5a-c).

#### Spleen

**Fig. 6:**
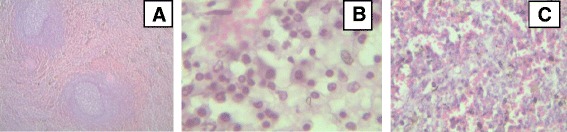
Major histopathological findings in spleen of cattle experimentally infected with *T. vivax* isolates. **a** Haemosiderosis and white pulp with dark zone (low magnification); **b** erythrophagocytosis (high magnification); **c** spleen with severe lymphoid proliferation forming lymphoid follicles in the red zone (low magnification). (haematoxylin and eosin staining)

Lymphoid hyperplasia in the white and red pulp of spleen was the common microscopic lesion in spleen of infected cattle. In some calves lymphoid depletion with formation of cavities at the center of lymphoid follicles in the white pulp, and haemosiderosis were detected. The red pulp of the spleen showed lymphoid hyperplasia with formation of follicles dominated by lymphoblasts (Fig. 6a-c).

### Liver

**Fig. 7:**
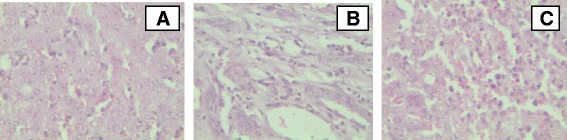
Major histopathological findings in liver of cattle experimentally infected with *T. vivax* isolates. **a** Necrosis of hepatocytes, portal tract and bile duct proliferation, plasma cell infiltration and hemorrhages (low magnification); **b** liver showing centrilobular hepatocyte necrosis; most hepatocytes in affected area have condensed (pyknotic) nuclei (low magnification); **c** hepatitis with severe accumulation of lymphocytes and plasma cells, proliferation of biliary ducts and dilation of the vessels (low magnification), (haematoxylin and eosin staining)

The major microscopic changes in the liver of the infected animals were severe zonal hepatic necrosis especially of the centrilobular and periportal regions. Hepatocytes at the center of necrotic areas were either totally lysed with karyorrhectic nuclei or with condensed pykinotic nuclei. A heavy infiltration of mononuclear leukocytes and lymphocytes in the portal areas of the liver was detected. In addition, histopathological examination of the liver revealed dilated blood vessels filled with proteinaceous hyaline membranes and fibrin deposits (Fig. **7**a-c).

### Brain

**Fig. 8:**
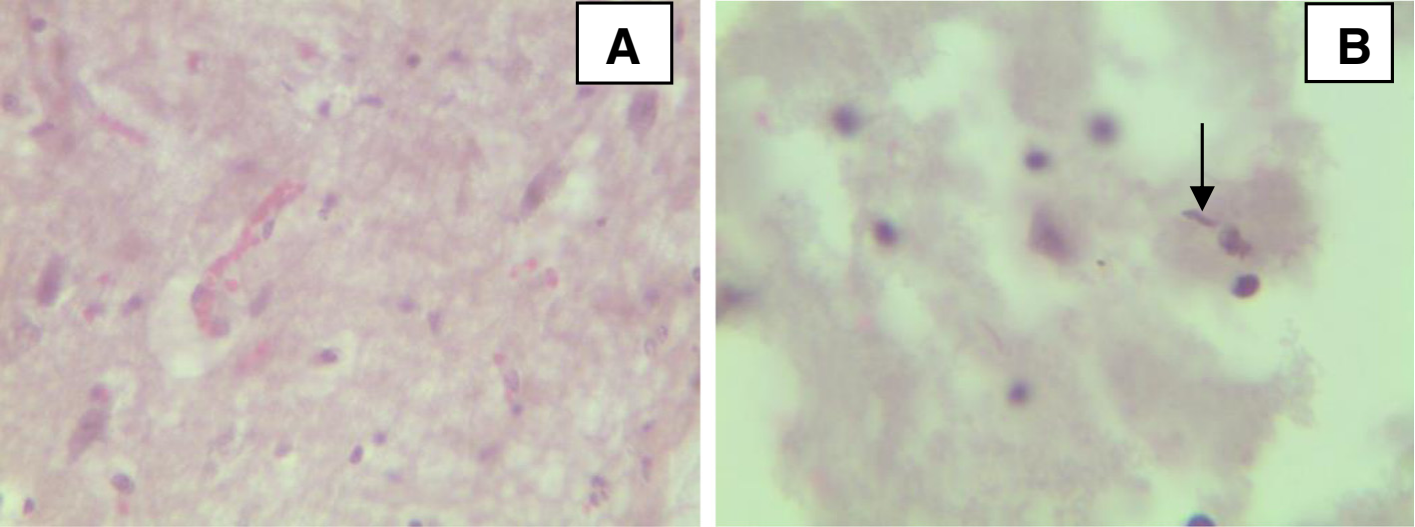
Major histopathological findings in brain of cattle experimentally infected with *T. vivax* isolates. **a** Haemorrhages and congestion with neuronal necrosis characterized by angular and shrunken neuronal cell bodies, increased gliosis and small glial nodules (high magnification); **b** oedema of brain with infiltration of plasma cells and presence of parasites (arrow) (high magnification), (haematoxylin and eosin staining)

In almost all infected animals a meningoencephalitis of varying severity was detected. The brain showed neuronal necrosis with shrunken angular neuron and pyknotic somas. In some brains with ischemic type neuronal necrosis gitter cells and small glial nodules (neuronophagia) were present. Astrogliosis with hypertrophy of astrocytes, perivasculatis with mononuclear cuffs and focal gliosis were also detected in infected brains. In some animals hypertrophy of the vascular endothelium, and an accumulation of a few lymphocytes and macrophages around the affected vessels were present (Fig. 8a-b).

## Discussion

All the infected cattle developed parasitaemia at 6 dpi in NT group and at 12 dpi in TT group. Cattle infected with non-tsetse infested *T. vivax* isolate revealed a more rapid parasitaemia compared to those infected with tsetse infested isolate within a week post infection. This might be associated with the fact that artificial intravenous inoculation mimics the mechanical transmission of *T. vivax* in the non-tsetse adapted isolates compared to that of the tsetse adapted isolate. Further explanation for the early onset of parasitaemia in the NT infected cattle could be the increased growth rate of the NT parasite. This result is supported by findings obtained with *T. brucei* [21] infections; when this parasite is syringe passaged in rodents an increase in parasitaemia and virulence can be detected. This has been attributed to a lack of a reset when going through the vector. In the current work the early appearance and peak parasitaemia in the NT isolates seems not to be related to a higher virulence in the NT compared to TT isolates.

The present finding roughly agrees with the findings of Adeiza et al. [22] who reported a mean of 5.3 days pre-patent period for *T. vivax* infected goats. However Osman et al. [23] reported pre-patent periods of 4.20 ± 1.64 and 4.33 ± 2.31 days for *T. vivax* infected Nubian and Nilotic dwarf goats respectively. The pre-patent period of infection by *T. vivax* is variable, depending on the immune status of the host, virulence of the parasite isolate and the infective dose [11, 24]. The typical signs of clinical trypanosomosis were observed in both groups of the infected animals. The results revealed that the beginning of pyrexia corresponds with the initial parasitaemia. Both of these parameters follow the same progression over time and reach peak within the first three weeks p.i., showing eventually a reduction. Similar findings were reported by Adeiza et al. [22] in Savannah brown goats experimentally infected with *T. brucei* and *T. vivax.*

In the present study significant decrease in mean PCV and differential WBC counts were observed in all infected groups compared with the non-infected control group. These results were supported by the findings of Maxie et al. [25] which detected pancytopenia, i.e., anaemia, leukopenia, and thrombocytopenia associated with *T. vivax* and *T. congolense* infections of cattle. Decrease in mean PCV values might be correlated with the decrease in total RBC count. A relative deficiency of blood cells occurs initially due to haemodilution and further exacerbated by haemolytic anaemia. Hemolysis could be caused by mechanical injury to erythrocytes by the lashing action of the powerful locomotory flagella and microtubule-reinforced bodies of the high number of the organisms during parasitaemia [26]. Erythrocyte membrane damage has also been associated with adhesion of erythrocytes and reticulocytes to trypanosome surfaces via sialic acid receptors leading to damages to erythrocyte cell membranes [27]. An increase in body temperature was reported to decrease erythrocyte plasticity and longevity *in-vivo* [28]. An increase in body temperature also increases the rate of immunochemical reactions thereby initiating lipid peroxidation of erythrocytes [1]. Our result is in line with these findings, as sharp decline in PCV occurred during the first month of the study when parasitaemia and pyrexia were high. During this period the huge number of parasites and high body temperature may contribute to the severity of anaemia. Furthermore, living and dead trypanosomes can produce various forms of active chemical substances, which can elicit erythrocyte injury [29, 30].

The present study showed that the mean differential WBC count of infected groups is significantly lower than that of non-infected control group. This was in line with the findings of Maxie et al. [25] which observed leukopenia in *T. vivax* and *T. congolense* infections of cattle, and Allam et al. [31] in *T. brucei* infected gilts. The lower counts of lymphocytes and neutrophil observed in the infected groups may be attributed to the immunosuppressive actions of trypanosome infection [32]. The increase in relative number of monocytes in infected groups is supported by the findings of Silva et al. [12] who showed that the leukocyte changes are characterized by relative lymphocytosis and monocytosis, and decrease in the neutrophil counts. The relative rise in monocyte count in the present study may be explained by its role in the destruction of numerous damaged erythrocytes. The eosinopenia noticed in this study was also observed in *T. brucei* infected gilts [31], and in goats and sheep infected with *T. vivax* [33].

In the current study significant gross lesions and histopathological changes were observed in the lymph nodes, spleen, liver, lung, heart, kidney, small intestine and brain in infected animals, whereas the gross and histopathological examination of tissue sections from positive control animals showed no visible area of lesions. Similar pathological findings were reported by several authors in trypanosome infections [34–40]. In infected groups gross distortion of tissue architecture with complete loss of cellular morphology marked by pronounced inflammatory changes including enlarged lymph nodes, hepatomegaly, splenomegaly, edematous and haemorhagic lesions on the small intestine and brain were found. In addition, the histopathological investigation in this study showed that *T. vivax* can localize extra-vascularly in tissues and produce lesions. The pathological lesions observed in the tissues of the infected animals are in agreement with Opara and Fagbemi [41], who reported organ degenerative changes in animal trypanosomiasis. Similar to our finding splenomegaly and hepatomegaly were reported by Fatihu et al. [37] in experimental infected goats with *T. vivax*. The various forms of congestion and necrosis observed were also in consonance with findings of Archivio et al. [39] and Silva et al. [40].

The lesions in the lymph nodes of trypanosome infected animals included increased presence of plasma cell populations, and the perivascular mononuclear cell infiltration, mainly of lymphocytes, plasma cells and macrophages, hepatocyte degeneration and hyperplasia in the liver were the common feature in the present findings. These changes accord to the findings described by Omotainse and Anosa [42] in ovine infected with *T. vivax*, *T. congolense* and *T. brucei*. This might have played a significant role in the inability of the animals to successfully recover from the anaemia experienced in trypanosomosis [42].

In this study, hyperplasia of the red pulp, enlargement of the lymphoid nodules and proliferation of plasma cells were the lesions seen in the spleen in all infected groups is in agreement with the finding of Omotainse and Anosa [42]. Neuronal necrosis with shrunken angular neuron, pyknosis, perivasculatis, mononuclear cell infiltration like lymphocytes, plasma cells and macrophages were the lesions seen in the brain which might be associated the presence of parasites in the brain tissue (Fig. 8b) and encephalopathy as result of hypoglycemia due to reduction of glucose level [43]. Moreover, four infected animals in the current study displayed nervous signs were euthanized suggest the development of encephalopathy associated with hypoglycemia and or damage to brain tissue by the parasite. The findings of nervous signs in the present study could be the possible crossing of brain by *T. vivax* as it was reported previously in some species of trypanosomes such as *T. brucei, T. evansi* and *T. vivax* [38]. Nervous system lesions caused by these trypanosomes has been associated to the presence of trypanosomes in the nervous tissues [44–46]. Similarly in a study after *T. vivax* infection in Brazilian semiarid region demonstrated that *T. vivax* can cause nervous signs due to inflammatory and degenerative brain lesions in cattle [36]. Similar to the present study many necrotic foci in lymphoid and non-lymphoid organs with extravasated blood cells and trypanosomes in hemorrhagic spots were reported by Archivio et al. [39]. Most importantly, the infection resulted in multifocal lesions in the central nervous system along with vasogenic edema and damaged blood vessels characteristic of the late-stage ischemic necrosis caused by the wild-type strain [39].

## Conclusions

Young zebu cattle experimentally infected with *T. vivax* isolates from tsetse and non-tsetse infested areas developed acute form of trypanosomosis which was characterized by reduced feed intake, parasitaemia, pyrexia, enlarged prescapular lymph nodes, occasional diarrhea and oedema, anaemia, and leukopenia. Parasitaemia of the non-tsetse adapted isolates reached its peak early than that of the tsetse adapted isolate. Both infected groups resulted pathological lesions in various body systems and major organs. Consequently, all infected animals were susceptible to *T. vivax* infection showing variable clinical and pathological changes, manifested by severe responses.
